# Extracts from Field Margin Weeds Provide Economically Viable and Environmentally Benign Pest Control Compared to Synthetic Pesticides

**DOI:** 10.1371/journal.pone.0143530

**Published:** 2015-11-23

**Authors:** Prisila Mkenda, Regina Mwanauta, Philip C. Stevenson, Patrick Ndakidemi, Kelvin Mtei, Steven R. Belmain

**Affiliations:** 1 Nelson Mandela African Institution of Science and Technology, Arusha, Tanzania; 2 Jodrell Laboratory, Royal Botanic Gardens, Kew, Richmond, Surrey, United Kingdom; 3 Natural Resources Institute, University of Greenwich, Chatham Maritime, Kent, United Kingdom; United States Department of Agriculture, Beltsville Agricultural Research Center, UNITED STATES

## Abstract

Plants with pesticidal properties have been investigated for decades as alternatives to synthetics, but most progress has been shown in the laboratory. Consequently, research on pesticidal plants is failing to address gaps in our knowledge that constrain their uptake. Some of these gaps are their evaluation of their efficacy under field conditions, their economic viability and impact on beneficial organisms. Extracts made from four abundant weed species found in northern Tanzania, *Tithonia diversifolia*, *Tephrosia vogelii*, *Vernonia amygdalina* and *Lippia javanica* offered effective control of key pest species on common bean plants (*Phaseolus vulgaris*) that was comparable to the pyrethroid synthetic, Karate. The plant pesticide treatments had significantly lower effects on natural enemies (lady beetles and spiders). Plant pesticide treatments were more cost effective to use than the synthetic pesticide where the marginal rate of return for the synthetic was no different from the untreated control, around 4USD/ha, compared to a rate of return of around 5.50USD/ha for plant pesticide treatments. Chemical analysis confirmed the presence of known insecticidal compounds in water extracts of *T*. *vogelii* (the rotenoid deguelin) and *T*. *diversifolia* (the sesquiterpene lactone tagitinin A). Sesquiterpene lactones and the saponin vernonioside C were also identified in organic extracts of *V*. *amygdalina* but only the saponin was recorded in water extracts which are similar to those used in the field trial. Pesticidal plants were better able to facilitate ecosystem services whilst effectively managing pests. The labour costs of collecting and processing abundant plants near farm land were less than the cost of purchasing synthetic pesticides.

## Introduction

Tanzania is among the top twenty largest producers of common beans, *Phaseolus vulgaris* L., in the world, and is the second largest producer after Kenya in sub-Saharan Africa.[1] Common beans are rich in protein and are a good source of several nutrients that are considered key elements for mental development.[2,3] Insect pests are one of the most common factors affecting production of beans which particularly affect production in Tanzania where the average bean yield was 884 kg/ha in 2013 in comparison to average global yields of 1427 kg/ha.[4] Due to the severity of different insect pests affecting beans, many African farmers increasingly resort to frequent use of commercial synthetic pesticides.[5] Such pest management practices are increasingly criticised as unsustainable and difficult to incorporate into agro-ecological intensification programmes aimed at developing sustainable agricultural practices and promoting ecosystem services.[6–8]

Plants with pesticidal properties have been investigated for decades as alternatives to synthetics, but little progress has been made to develop new products.[9,10] Although research on pesticidal plants is increasing, it is failing to address gaps in our knowledge that constrain their adoption.[11] One of these gaps is their evaluation under realistic field conditions to assess their efficacy as well as whether their use can be economically beneficial to farmers. In comparison to concentrated synthetic products, pesticidal plants should be more environmentally benign due to their short persistence, naturally low concentrations of a more diverse suite of active ingredients and anti-feedant/repellent modes of action. Although there are some studies highlighting the relative benefits of pesticidal plants for ecosystem services, such as increased biological control,[12] there are relatively few studies which provide comparative evidence of ecosystem impact of synthetics and pesticidal plants under field conditions.[13] Thus the aims of our study are to: 1) investigate optimal application methods of pesticidal plants, particularly weed species that are widely available and abundant in bean production ecosystems, for insect pest control on common bean; 2) compare the effects of a common synthetic pesticide and pesticidal plants on the level of pest control and their potential effects on predatory insect species; and 3) a cost-benefit analysis on these pest management options.

## Materials and Methods

### Study site

The study was conducted at Lyamungo, Moshi, Tanzania (Latitude 3°13’59.59”S Longitude 37°14’54”E) during the main cropping season (March-June 2014). The study was carried out on private land, the owner of the land, Tanzania Coffee Research Institute, gave permission to conduct the study on this site. The site is at an elevation of 1268m asl. The mean annual rainfall is 1200mm with the mean maximum temperature of 21.7°C and the mean minimum temperature of 13.6°C.

### Experimental design

The field was disc harrowed and ridged prior to planting. The common bean (*Phaseolus vulgaris*) seeds used for planting were of the variety Lyamungo 90 and obtained directly from the breeder at Selian Agricultural Research Institute, Tanzania. The seeds were planted at a spacing of 50 cm between rows and 20 cm within rows in 3 x 4 m plots which were 1 m apart. Three seeds were seeded per hill and then thinned to two plants. Diammonium phosphate fertilizer was applied according to manufacturer’s instructions during planting of the seeds. The experimental layout was a randomized complete block design, and the treatments were replicated on four blocks, all within the same field location.

### Plant species collection and processing

Fresh leaves of *Tephrosia vogelii* (Hook f.), *Vernonia amygdalina* (Delile), *Lippia javanica* (Burm.f.) Spreng. and *Tithonia diversifolia* (Hemsl.) A. Gray were collected from different locations around Arusha and Moshi (voucher specimens and GPS coordinates lodged at Nelson Mandela African Institution of Science and Technology, Arusha, Tanzania). These four species were chosen due to their wide abundance around farms, roadsides and bushland, their familiarity to farmers and considerable existing knowledge on their efficacy, bioactive constituents and safety.[14–29] To ensure uniformity, the leaves from each collection were mixed together for each species before drying. Leaves were dried under shade for a week and then crushed using a mill and sieved into a fine powder. Powders were stored in black plastic bags in dark, dry conditions until required.

### Chemical analysis

Leaf material of *T*. *vogelii*, *T*. *diversifolia* and *V*. *amygdalina* was analysed using a Waters Alliance LC system with a ZQ LC-MS detector on a Phenomenex Luna C18(2) column (150 × 4.0 mm i.d., 5 μm particle size) operating under gradient conditions, with A = MeOH, B = H_2_O, C = 1% HCO_2_H in MeCN; A = 0%, B = 90% at t = 0 min; A = 90%, B = 0% at t = 20 min; A = 90%, B = 0% at t = 30 min; A = 0%, B = 90% at t = 31 min; column temperature 30°C and flow rate of 0.5 ml/min as described previously.[22] For *T*. *vogelii* this ensured the pesticidal chemotype was used for field experiments.[20] Aliquots (10μL) of a filtered (0.45um) methanol extract (5% w/v) and a filtered water extract (5% w/v) were injected directly on to the column and compared with laboratory libraries of rotenoids, flavanol and flavones as reported earlier.[22] Extracts of *T*. *diversifolia* and *V*. *amygdalina* were analysed by LC using High Resolution Electrospray Ionisation Mass Spectroscopy (HRESIMS) which facilitated the tentative identification of previously described compounds that are associated with the biological activity. HRESIMS data was recorded using a Thermo LTQ-Orbitrap XL mass spectrometer hyphenated to a Thermo Accela LC system performing chromatographic separation of 5 μl injections on a Phenomenex Luna C18(2) column (150 mm × 3.0 mm i.d., 3 μm particle size) with a linear mobile phase gradient of 10–100% aqueous MeOH containing 0.1% formic acid over 20 min. Spectra were recorded in either positive or negative modes at 30,000 resolution.

*Lippia javanica* is also known to vary chemically by season and geographic location and was analysed as described previously[30] by collecting volatiles from dry powdered leaves onto a Solid Phase Micro Extraction (SPME) fibre, coated with polydimethylsiloxane/divinylbenzene (Supeclco) for 5 minutes and desorbing directly onto an Agilent 6890 gas chromatograph coupled to an Agilent 5973 mass spectrometer with a DB-5 fused silica capillary column (30 m length, 0.25 mm diameter, 0.25 μm film thickness, (Agilent). Desorption was splitless with helium at a constant flow rate of 1ml/min as a carrier gas. The column temperature was held at 60°C for 2 minutes, then programmed to 240°C at 6°C/min. The ion source was held at 150°C, and the transfer line was held at 250°C.

### Field treatments

To determine potential concentration effects, two different concentrations of each plant species were made, 10% and 1% w/v. As extractions were carried out in water, a second variable of adding 0.1% soap during or after extraction was also included. Soap was added as it is known to increase extraction of non-polar compounds and acts as a surfactant during application.[20] Thus for each plant species, there were four treatments (1 and 10% w/v, with and without soap during extraction), each replicated four times, thus giving 16 blocks per plant species. In making all extracts, the correct amount of plant powder was weighed and added to water to extract at ambient temperature (20±5°C) for 24 hours. Extracts were kept in 10 l buckets with lids in the shade and filtered through a fine cloth to remove all plant material that may inadvertently clog the sprayer.

In order to account for the different parameters, negative controls in the trial consisted of water + 0.1% soap, water only and an untreated control. The positive control in the trial was synthetic pesticide Karate (lambda-cyhalothrin pyrethroid, Syngenta) which was applied as per the manufacturers’ instructions. All controls were replicated across four blocks. All treatments were sprayed throughout the growing season at an interval of 7 days starting one week after bean plant emergence. A 15-litre knapsack sprayer was used to apply the various treatments, and the sprayer was thoroughly cleaned with soap and water prior to being re-filled with another formulation for application.

### Sampling for insect pest infestation

All assessments were carried out the day before treatments were to be sprayed. The target insect pests to be evaluated were aphids (*Aphis fabae*), bean foliage beetle (*Ootheca mutabilis* and *O*. *bennigseni*) and flower beetle (*Epicauta albovittata* and *E*. *limbatipennis*). Three inner rows from each plot were selected for sampling. Ten plants in the selected three middle rows were counted and visually examined to record the number of plants infested by each insect pest, thus providing the percentage of plants infested (incidence). The black bean aphid, *Aphis fabae*, was observed on bean plants for 10 weeks. Due to often very high numbers, a categorical index was used to assess aphid abundance, where 0 = None; 1 = A few scattered individuals; 2 = A few isolated colonies; 3 = Several isolated colonies; 4 = Large isolated colonies; and 5 = Large continuous colonies. Aphid damage was defined as wilted or blackened leaves (due to honeydew accumulation). The abundance of foliage beetle and flower beetle was determined by counting the total number. Field observations of bean foliage beetle and flower beetle were conducted during the 1st to 4th week and 5th to 8th week, respectively, after bean emergence. Two species of foliage beetle are known to be present in the Kilimanjaro region of Tanzania, *Ootheca mutabilis* and *O*. *bennigseni*.[31] As they cause similar damage and are not easy to tell apart in the field, we did not attempt to identify their presence to the species level and recorded the total number of foliage beetle found during surveys. Ootheca damage is distinct, causing holes in the middle of leaves, and is easily recognised from other insect damage. The most common blister beetles in Tanzania are *Epicauta albovittata* and *E*. *limbatipennis*;[32] however, there are many similar-looking species causing similar damage, and we did not attempt to identify them to species level. Locally, they are called flower beetles as the adults commonly eat the flowers of all pulse crops and other vegetables, again causing quite distinct damage at the flowering stage. The severity or degree of infestation in each infested plant was assessed by scoring the extent of damage using grades, where 0 = No damage; 1 = Showing damage up to 25%; 2 = Damage from 26%-50%; 3 = Damage from 51%-75% and 4 = Damage more than 75%. The abundance of lady beetles (adults and larvae) (Coccinellidae) and spiders (Araneae) were counted at each assessment period from their first appearance.

### Data Analysis

Differences among treatments in insect incidence, abundance, damage and bean yield were assessed by analysis of variance (ANOVA) and Tukey’s post-hoc Honestly Significant Difference (HSD) test to separate the means at the 95% confidence interval. Analyses were performed in XLSTAT version 2015.1.01 (Addinsoft, Paris, France).

Grain yield higher than obtained in the negative control plots was assumed to be solely due to pesticide application. An economic analysis according to Ndakidemi[33] was carried out by computing the profit or Marginal Net Return (MNR) for each treatment using the formula:

MNR=Y×P−TVC

Where

MNR = Marginal Net Return (Profit)

Y = is grain yield (kg/ha)

P = is selling price of common beans at harvest (USD/kg)

TVC = the total variable cost, i.e. cost of inputs and labour charges (e.g. seeds, pesticide, labour for planting, weeding, pesticide application) as shown in Table 1.

**Table 1:** Total variable costs (TVC) used in economic analysis of profit from different treatments applied to common bean plants.

Input/activity	Total cost (USD/ha)
Seeds 24 kg @1.515USD	36.36
Fertilizer 12 kg @ 0.85	10.18
Soap, 5 litres @2.18 USD	10.91
Synthetic (Karate), 6 litres @ 9.09 USD	54.55
Collection of pesticide leaves	18.18
Grinding of leaves	9.09
Land preparation	72.73
Planting and fertilizer application	36.36
Weeding	60.60
Labour for pesticide preparation and application	36.36
Harvesting	36.36

Partial budgeting was used to estimate the profit per hectare for each treatment. The profit was estimated by deducting the total variable cost from the income derived from the yield.

Furthermore, the marginal rate of return (MRR) for each treatment was derived from

MRR=MNR/TVC

Where

MRR = Marginal rate of return

## Results and Discussion

### Arthropod presence and bean plant damage levels

The effects of the four plant species extracts were not observed to have any statistical difference when comparing the application rates of 1 and 10 percent (w/v) in terms of insect abundance, incidence, damage or any observed difference in overall bean yield (Table 2). Also contrary to expectations, no statistical significance was observed in the results with respect to whether soap was added during the extraction process or after extraction (Table 2). Furthermore, no statistical difference was observed among the three negative controls (water+soap, water only, untreated) with respect to insect abundance, incidence, damage and bean yield (Table 2). The lack of difference among these parameters facilitated the pooling of treatment data to compare each plant species against a single negative and positive control treatment.

**Table 2:** Analysis of variance (ANOVA) on the average abundance, incidence and damage by key pests found on common bean plants and total grain yield, comparing three control treatments (untreated, water+soap, water only), two concentration levels (1%, 10%) and when soap was added (during extraction, after extraction). In all cases, there were no significant differences across parameters at the 95% confidence interval using Tukey’s post-hoc Honestly Significant Difference (HSD) test.

	Insect abundance			Plants infested (% incidence)			Index of plant damage			Grain yield
Treatment	Aphid	Foliage beetle	Flower beetle	Aphid	Foliage beetle	Flower beetle	Aphid	Foliage beetle	Flower beetle	
Untreated	2.87	1.68	2.56	30.25	22.81	25.31	2.48	2.25	2.09	1193.21
Water + soap	3.10	1.56	2.50	29.00	24.39	26.50	2.40	2.16	1.75	1207.50
Water only	2.65	1.37	2.31	26.75	20.97	25.20	2.23	2.00	2.00	1205.69
F	0.42	0.78	0.19	0.42	0.31	0.12	0.42	0.54	1.55	0.05
Pr > F	0.66	0.47	0.83	0.66	0.74	0.89	0.82	0.74	0.24	0.95
1% concentration	1.29	1.05	1.27	12.38	15.94	13.36	0.99	0.83	1.42	1776.64
10% concentration	1.40	1.04	1.27	12.19	15.23	13.13	0.84	0.84	1.30	1656.97
F	0.30	0.00	0.00	0.01	0.47	0.02	1.09	0.02	2.39	2.22
Pr > F	0.58	0.95	0.96	0.94	0.50	0.90	0.30	0.90	0.13	0.14
Soap during extract	1.38	1.02	1.28	12.38	15.86	12.97	0.96	0.82	1.33	1732.40
Soap after extract	1.31	1.07	1.26	12.19	15.31	13.52	0.88	0.85	1.39	1701.20
F	0.13	0.20	0.02	0.01	0.28	0.09	0.37	0.07	0.58	0.15
Pr > F	0.72	0.65	0.89	0.94	0.60	0.77	0.55	0.79	0.45	0.70

**Fig 1:**
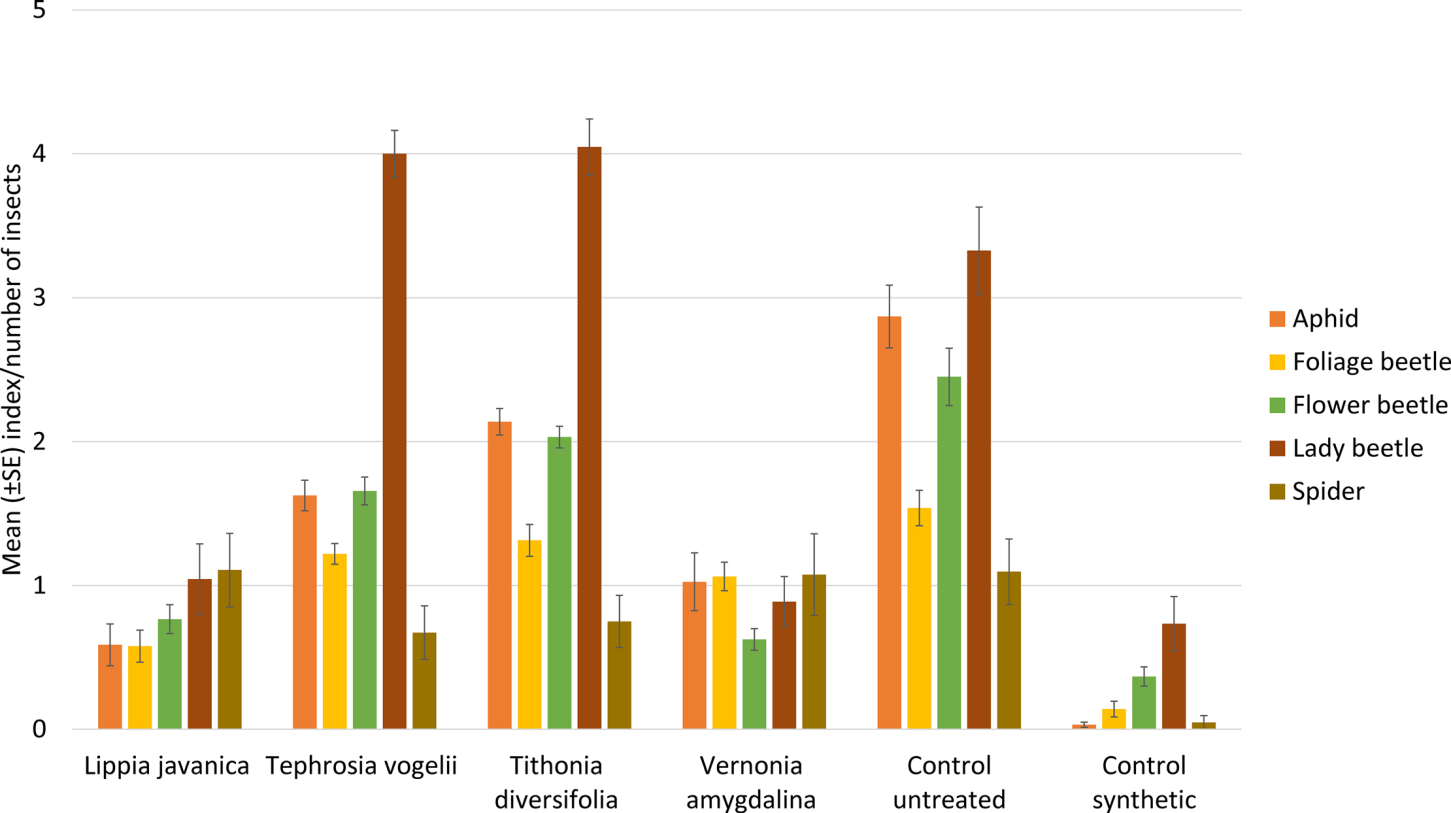
Abundance of key pests and predators on bean plants. Aphid abundance uses a 1–5 severity index, whereas all other insects are counted.

The mean abundance of aphids, foliage beetle, flower beetles, lady beetles and spiders were shown to significantly vary across the treatments (Fig 1 and Table 3). The synthetic pesticide was superior in reducing abundance of all pest insects in comparison to the plant extracts with the exception of *V*. *amygdalina* which provided comparable control of flower beetles as the synthetic. All four plant species were able to significantly reduce abundance of aphids and flower beetles in comparison to the untreated control; whereas *V*. *amygdalina* and *L*. *javanica* were the only plant treatments able to reduce the abundance of foliage beetle (Table 3). Although *Tephrosia vogelii* and *Tithonia diversifolia* were the least effective of the four plant species evaluated, they were the most benign in terms of impact on predators, showing no significant impact on lady beetle abundance. All four plant species had no effect on the abundance of spiders, whereas the synthetic pesticide treatment significantly reduced both lady beetle and spider abundance (Table 3).

**Table 3:** Analysis of variance (ANOVA) on the average abundance of key pests and predators and the average incidence and damage of key pests found on common bean plants sprayed weekly with extracts of four plant species and positive/negative control treatments. Values in the same column followed by the same letter are not significantly different from each other at the 95% confidence interval using Tukey’s post-hoc Honestly Significant Difference (HSD) test.

	Insect abundance					Plants infested (% incidence)			Index of plant damage		
Treatment	Aphid	Foliage beetle	Flower beetle	Lady beetle	Spider	Aphid	Foliage beetle	Flower beetle	Aphid	Foliage beetle	Flower beetle
Control -	2.87 ^a^	1.54 ^a^	2.45 ^a^	3.33 ^a^	1.10 ^a^	28.60 ^a^	22.51 ^a^	25.63 ^a^	2.36 ^a^	2.01 ^a^	1.99 ^a^
Control +	0.03 ^d^	0.14 ^d^	0.37 ^d^	0.73 ^b^	0.05 ^b^	0.56 ^c^	3.75 ^d^	3.52 ^c^	0.02 ^e^	0.39 ^c^	0.52 ^c^
*Lippia javanica*	0.59 ^c^	0.58 ^c^	0.77 ^c^	1.04 ^b^	1.11 ^a^	4.25 ^c^	12.81 ^c^	6.88 ^c^	0.71 ^cd^	0.30 ^c^	1.45 ^b^
*Tephrosia vogelii*	1.63 ^b^	1.22 ^a,b^	1.66 b	4.00 ^a^	0.67 ^a,b^	18.00 ^b^	16.09 ^b,c^	17.50 ^b^	1.04 ^b,c^	0.94 ^b^	1.19 ^b^
*Tithonia diversifolia*	2.14 ^b^	1.31 ^a,b^	2.03 ^a,b^	4.05 ^a^	0.75 ^a^	22.63 ^b^	18.59 ^a,b^	21.88 ^a,b^	1.44 ^b^	1.09 ^b^	1.45 ^b^
*Vernonia amygdalina*	1.03 ^c^	1.06 ^b^	0.63 ^c,d^	0.89 ^b^	1.08 ^a^	4.25 ^c^	14.84 ^b,c^	6.72 ^c^	0.49 ^d^	1.02 ^b^	1.34 ^b^
F	78.96	46.48	77.03	66.00	7.04	137.10	47.19	87.01	80.85	64.99	58.45
Pr > F	0.0001	0.0001	0.0001	0.0001	0.0001	0.0001	0.0001	0.0001	0.0001	0.0001	0.0001

**Fig 2:**
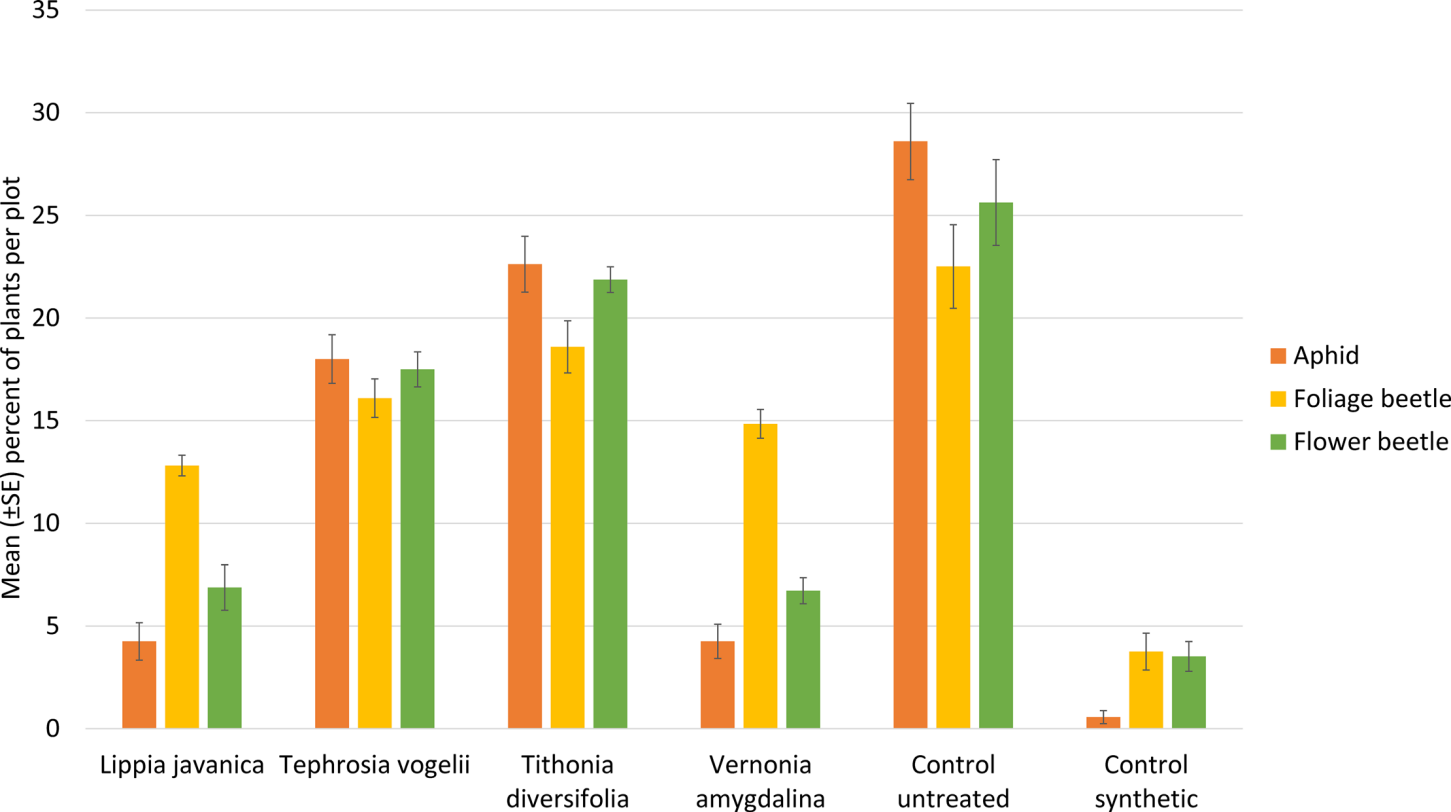
Percentage of bean plants infested with key pest species.

The percentage of bean plants infested with the three pest species varied from approximately 5 to 20 percent across the four plant species treatments (Fig 2). This is significantly less than the untreated control infestation rate of 25 to 30 percent of bean plants infested, but higher than the synthetic pesticide treatment where infestation was below five percent. *L*. *javanica* and *V*. *amygdalina* were as effective as the synthetic control in reducing the percentage of plants affected with aphids and flower beetles (Table 3). All four plant species were significantly better than the untreated control with the exception of *T*. *diversifolia* which had no effect on the incidence of foliage beetle and flower beetle.

**Fig 3:**
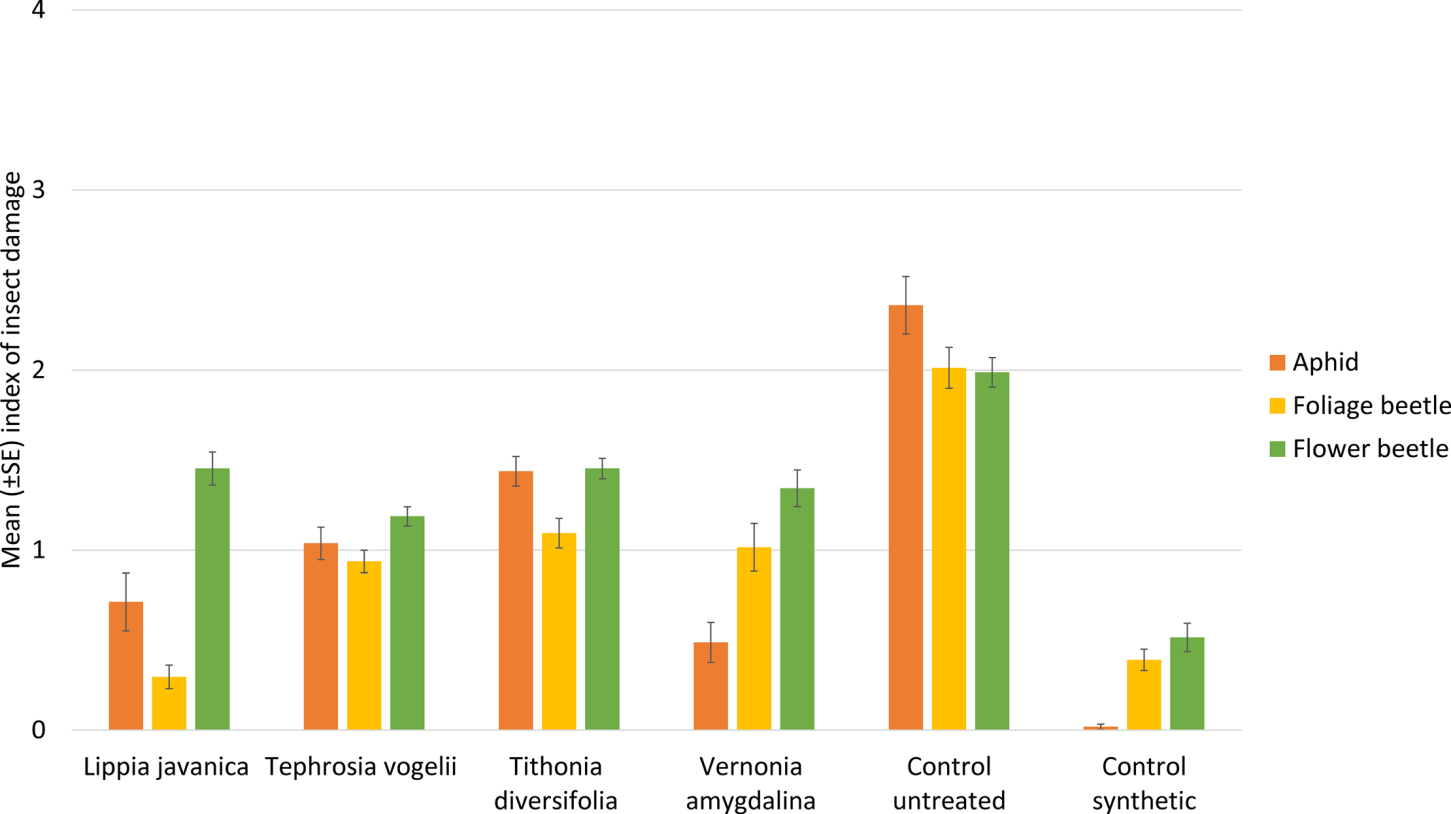
Insect damage to bean plants by key pest species affecting beans. Data are expressed as an index where 0 = No damage; 1 = Damage up to 25%; 2 = Damage 26%-50%; 3 = Damage 51%-75%; and 4 = Damage 76%-100%.

Insect damage to bean plants was reduced by all four plant species treatments in comparison to the untreated control; whereas the synthetic treatment was the most effective of all treatments in reducing damage (Fig 3). However, *L*. *javanica* was comparable to the synthetic in reducing damage caused by foliage beetle (Table 3). *V*. *amygdalina* and *L*. *javanica* were the most effective plant species treatments to reduce damage caused by aphids.

### Bean yield and cost-benefit analysis

The negative control treatment resulted in significantly lower average numbers of pods per plant, seeds per pod, weight of seeds and overall yield when compared to the synthetic and plant species treatments (Table 4). The overall yield was highest when using *T*. *vogelii* followed by *T*. *diversifolia*, *V*. *amygdalina*, synthetic control and *L*. *javanica*, with the untreated control having the lowest yield. Although there were some significant differences in the number of seeds per pod and average grain weight, it appears the main parameter explaining total yield was the number of pods per plant. This trend is not entirely consistent, and, for example, *V*. *amygdalina* has the lowest number of pods per plant out of the plant species treatments, but also has the highest average seed weight thus presenting an overall yield statistically similar to the synthetic and other plant species treatments.

**Table 4:** Analysis of variance (ANOVA) on the yield and economic return of common bean plants sprayed weekly with extracts of four plant species and positive/negative control treatments. Values in the same column followed by the same letter are not significantly different from each other at the 95% confidence interval using Tukey’s post-hoc Honestly Significant Difference (HSD) test.

Treatment	Pods per plant	Seeds per pod	100 grain weight (g)	Overall yield (kg/ha)	Marginal net return (USD/ha)	Marginal rate of return (USD/ha)	Percent increase over control
Control -	3.49 ^d^	2.31 ^c^	55.63 ^c^	1201.92 ^c^	1136.66 ^c^	4.06 ^c^	-
Control +	6.12 ^b^	3.54 ^a^	60.19 ^b^	1578.48 ^b^	1483.65 ^b^	4.06 c	30.5
*Lippia javanica*	5.69 ^b,c^	3.56 ^a^	60.45 ^b^	1424.25 ^b,c^	1408.30 ^b,c^	4.42 ^b,c^	23.8
*Tephrosia vogelii*	7.15 ^a^	3.74 ^a^	60.41 ^b^	1921.75 ^a^	2011.34 ^a^	5.62 ^a^	76.8
*Tithonia diversifolia*	7.44 ^a^	3.66 ^a^	60.56 ^b^	1835.50 ^a^	1906.79 ^a^	5.32 ^a,b^	67.6
*Vernonia amygdalina*	4.81 ^c^	3.06 ^b^	65.39 ^a^	1685.71 ^a,b^	1725.22 ^a,b^	5.50 ^a^	51.7
F	41.92	21.21	10.14	18.67	19.16	11.82	
Pr > F	0.0001	0.0001	0.0001	0.0001	0.0001	0.0001	

Although the synthetic treatment was generally more effective in managing insect pests than the four plant species treatments, there is generally little difference in terms of economic profit due to its higher input cost (Table 1). The synthetic and plant species treatments resulted in a higher marginal net return (Table 4) than the negative control. However, the synthetic treatment is generally no better than the negative control in terms of the marginal rate of return. The marginal rate of return with *T*. *vogelii*, *V*. *amygdalina* and *T*. *diversifolia* was higher than the positive and negative control treatments.

### Chemical analysis

As previously reported,[30] chemical analysis of *L*. *javanica* allowed the identification of several constituents in the volatile component. Compounds were identified by retention time and the MS spectrum as compared to data in the NIST library. The major component was identified as camphor which occurred along with minor components including camphene, α-pinene, eucalyptol, Z and E α-terpineol, linalool, cymene, thymol, 2-carene, caryophyllene and α-cubebene. Camphor has well-documented insecticidal properties and may account for the biological activity of this plant species in our study.[34] While these compounds are likely to be only sparingly soluble in water, in practise farmers use crudely filtered extracts that produce a suspension containing plant material, thus these components are likely to contribute to the biological effect of the extract in the field. Analysis of the methanol extract of *T*. *vogelii* confirmed the plant material used was the pesticidal chemotype 1[22] and contained the rotenoids deguelin, tephrosin and rotenone (with deguelin being the most abundant) but did not contain the obovatin-5-*O*-methylether or other related flavonoids previously identified in chemotype 2 which is reported to be inactive.[20] Water can extract rotenoids despite their low polarity, thus extracts used by farmers will contain these compounds.[19] HRESIMS data facilitated the identification of the major compounds in *T*. *diversifolia* methanol extract from the molecular ion in positive mode LC-MS [M+H]^+^ as the sesquiterpene lactones tagitinin A (RT = 13.75 min m/z = 369.19141) C_19_H_29_O_7_ tagitinin C (RT = 14.42 min m/z = 349.16678 C_19_H_25_O_6_). Both compounds were reported recently to be to be the major compounds in this species,[35] while other research indicated tagitinins to have insecticidal activity.[16] Accordingly, it is likely that the presence of these major compounds is responsible for the toxicity of this plant in the field trials, particularly since they also occurred in the water extracts, albeit at just 25% of the concentration at which they occurred in the methanol extracts.[36] Similarly, the main components of *V*. *amygdalina* were tentatively identified from the molecular ion in positive mode [M+H]^+^ as follows: vernodalin (RT 12.19 min m/z = 361.1302 C_19_H_21_O_7_), 11,13-dihydrovernodalin (RT 12.37 min. m/z = 363.1455 molecular formula C_19_H_23_O_7_) and vernonioside C (RT 18.93 min, m/z = 781.44373 C_41_ H_65_ O_14_); however, only the saponin occurred in water extracts and at a similar concentration to that occurring in methanol while vernodalin was absent and dihydrovernodalin only present in trace amounts. Like tagitinin A and C, the first two compounds are sesquiterpene lactones which have been shown to exhibit antimalarial, antibacterial and cytotoxic activities.[37,38] While no insect activity is yet reported for these compounds, sesquiterpene lactones are known for their potent anti-feedant and toxic activities and may contribute to the activity found in other systems with *V*. *amygdalina* if their extraction can be optimised.[39] Vernonioside is one of several steroidal saponins known from *V*. *amygdalina* which causes the leaves to taste bitter[40]. It is possible that these compounds exert similar repellent effects against insects. Furthermore, saponins have been known to cause toxicity to insects in other pesticidal plants.[41] Further work is required to establish the absolute role of each of these compounds.

Although the pesticidal plant treatments were applied at two different rates (1 and 10%w/v), no observable difference in effects were recorded with respect to insect incidence, abundance and damage nor with respect to bean yield in terms of number of pods, seeds per pod, seed weight and total yield. With an order of magnitude between the concentrations applied, it would be reasonable to expect some observable difference as such concentration effects are widely observed and supported.[42] However, due to the nature of the compounds being largely non-polar and the extraction solvent being water there may be a limit to the efficiency of compound extraction that peaks at 1%. There is evidence that adding soap during the extraction process facilitates the extraction of hydrophobic compounds in water.[20] Further research is clearly necessary to understand the limitations farmers face when using water as an extraction medium and how this can be optimised for non-polar plant compounds. It may be that soap does not improve extraction efficiency of all compounds or that specific soaps are more effective than others. Future research should chromatographically analyse extracts used in field work to inform the interpretation of results and a wider range of concentrations, particularly lower concentrations should be evaluated.

### Conclusions

Our study suggests that commonly available pesticidal plants in sub-Saharan Africa, often those considered as weeds and highly invasive, can be effectively used to control crop pests. The labour costs to collect and process such readily available plants does mean the farmer must consider the time inputs required to use them. However, with relatively inexpensive labour costs in most of sub-Saharan Africa, these costs are more affordable than using commercial synthetics. Particularly small scale farmers with limited income to buy pesticides will usually prefer to invest their labour as opposed to using cash inputs such as pesticides.[43,44] Our data suggest that these different input costs are what make using pesticidal plants more profitable than synthetics. So although the commercial synthetic generally performed better at controlling insects on common beans, the level of insect control was not vastly different from the plant pesticides. All the plant pesticides were still more effective than the control, and in some instances, were just as effective as the synthetic.

*T*. *vogelii* and *T*. *diversifolia* were generally less effective than *L*. *javanica* and *V*. *amygdalina* in reducing pest insect incidence, abundance and damage. Despite this, *T*. *vogelii* and *T*. *diversifolia* treatments produced significantly higher yields than all other treatments. The explanation for this may lie in fact that these two treatments were also observed to have the least impact on lady beetle and spider numbers. This could suggest a degree of compatibility where relatively more predation takes place on the bean plants treated with *T*. *vogelii* and *T*. *diversifolia*, with predators compensating for the lower pesticidal effect. The yield increase with *T*. *vogelii* and *T*. *diversifolia* was largely due to an increase in the number of bean pods per plant. Although common beans are generally self-pollinating, there is evidence that pollinators can increase bean yield.[45,46] Our study was not able to quantify pollinator visitation, although bees were clearly observed to visit bean flowers, and this may provide the explanation why *T*. *vogelii* and *T*. *diversifolia* had significantly higher numbers of pods if these treatments were generally more benign to pollinators as they were to predators. The *V*. *amygdalina* treatment was observed to have fewer pods per plant than the other treatments, whilst also having the highest seed weight. The higher seed weight is likely resultant from physiological compensation, as occurs with many plant species.[47,48] However, as *V*. *amygdalina* was particularly effective in reducing flower beetle incidence and abundance, the lower number of pods is unlikely due to higher flower damage; higher damage is not supported by our data. One possible explanation is an effect of the *V*. *amygdalina* treatment on pollination services leading to fewer successful fertilisation events. Further studies are required to understand the value of pest management strategies which can also protect/facilitate ecosystem services.

Our study used widely available, weedy plant species, which are relatively easy to collect and process without any danger of over-collection. Many other plant species with known pesticidal properties are not always abundant and can be remote from farm locations. Some pesticidal species are also more difficult to process, e.g. pyrethrum from *Tanacetum cinerariaefolium*[49] and azadirachtin from *Azadirachta indica*.[50] Higher costs of using rare or difficult-to-process plant species could change the economics of their use in favour of synthetics or more readily available plant species, even when such products may be relatively more effective. In conclusion, this field trial suggests using commonly available weeds with pesticidal properties can make both economic sense for farmers whilst also being less harmful to the environment and consumers. However, the results suggest there may be considerable insect species selectivity occurring with different plant species derived pesticides, and this merits further investigation in order to optimise ecosystem services and improve financial rates of return to farmers who choose to use pesticidal plants.
